# Increased risk of adverse gestational outcomes in pregnant women with primary Sjögren’s syndrome

**DOI:** 10.1136/rmdopen-2023-003616

**Published:** 2024-05-28

**Authors:** Zhen Tan, Meilin Shao, Yingbo Zhou, Li Wang, Yan Ma, Nan Xiang, Xiang Yuan, Bin Wang, Xiangliang Xie, Mingtao Zhou, Yumin Wang, Sidong Li, Xiaomei Li

**Affiliations:** 1 The First Affiliated Hospital of USTC, Hefei, Anhui, China; 2 Department of Physiology, Anhui Medical College, Hefei, Anhui, China; 3 Huainan First People's Hospital, Huainan, Anhui, China; 4 Ma'anshan People's Hospital, Maanshan, Anhui, China; 5 People's Hospital of Chizhou, Chizhou, Anhui, China; 6 Huangshan City People's Hospital, Huangshan, Anhui, China; 7 University of Science and Technology, Hefei, Anhui, China

**Keywords:** Sjogren's Syndrome, Autoimmunity, Risk Factors

## Abstract

**Objectives:**

This study aimed to identify risk factors contributing to diverse pregnancy outcomes in primary Sjögren’s syndrome (pSS) cases.

**Methods:**

A retrospective analysis was conducted on pregnant individuals with pSS, who received outpatient or inpatient care across multiple hospitals in Anhui Province, China, from January 2015 to December 2022.

**Results:**

This study included 164 pregnant women with pSS and 328 control subjects, with no statistically significant difference in average age between the two groups. Analysis of pregnancy outcomes revealed that, compared with the control group, pregnant women in the pSS group were more likely to experience miscarriages, both spontaneous (12.80% vs 1.52%, p<0.001) and therapeutic (6.10% vs 0.91%, p<0.05). The proportion of placental abnormalities detected during prenatal ultrasound in women from the pSS group was higher (14.63% vs 6.40%, p<0.05). In the analysis of pregnancy outcomes for live-born neonates, a higher incidence of congenital heart abnormalities was observed in the pSS group (27.34% vs 12.03%, p<0.05). While there were no significant differences between the pSS pregnancies in terms of both normal and adverse pregnancy outcomes, a comparison of fetal survival and fetal loss in pSS pregnancies revealed a greater use of prophylactic anticoagulant therapy in the fetal survival group. Notably, the application of low molecular weight heparin (LMWH) emerged as an independent protective factor for fetal survival.

**Conclusions:**

Compared with non-autoimmune controls, pregnancy in women with pSS presents more challenges. Importantly, we observed that the use of LMWH as anticoagulant therapy is an independent protective measure for fetal survival.

**WHAT IS ALREADY KNOWN ON THIS TOPIC**

- Pregnancy in women with primary Sjögren’s syndrome is a high-risk process.

**WHAT THIS STUDY ADDS**

- Prophylactic anticoagulant therapy showed a protective effect against fetal loss in primary Sjögren’s syndrome.
- Prophylactic anticoagulant therapy with low-molecular-weight heparin is an independent protective factor for fetal loss in primary Sjögren’s syndrome.

**HOW THIS STUDY MIGHT AFFECT RESEARCH, PRACTICE OR POLICY**

- Pregnancy with primary Sjögren’s syndrome needs more guidance from a physician.
- Timely prophylactic anticoagulant therapy can reduce the risk of fetal loss.

## Introduction

Primary Sjögren’s syndrome (pSS) stands as a persistent autoimmune ailment marked by the infiltration of lymphocytes into the exocrine glands, gradually diminishing their secretory function and even extending to the extraglandular, yielding systemic repercussions.^1^ Predominantly afflicting middle-aged and older adults, pSS constitutes the most prevalent autoimmune connective tissue malady, exhibiting a prevalence of 0.01%–0.05% and an estimated 1:14 ratio of occurrence in males to females.^2^ Systemic organ involvement materialises in roughly one-third to one-half of patients,^3^ impacting diverse bodily systems such as joint, muscles, the respiratory apparatus, digestive tract, kidneys, central and peripheral nerves and the circulatory system.^4^

During pregnancy, the maternal immune system must tolerate the semiallogeneic fetus. In many pSS cases, antibodies capable of crossing the placental barrier, such as anti-Ro/SSA antibodies, have been identified. Several studies have indicated that high titres of Anti-Ro/SSA antibodies can impact fetal cardiac development, thereby increasing the risk of adverse pregnancy outcomes (APO).^5–7^ A recent review further suggested an elevated risk of adverse maternal and fetal outcomes in pregnant women with autoimmune diseases, with a notably increased risk of miscarriage in women with Sjögren’s syndrome.^8^ Another meta-analysis demonstrated that the dynamic changes in pregnancy risks for women with autoimmune diseases depend on factors such as maternal disease activity, organ damage, autoantibody titres and therapeutic interventions.^9^

To date, research on pregnancy outcomes in pSS patients has been limited compared with other autoimmune diseases such as systemic lupus erythematosus (SLE). A study by De Carolis *et al*,^10^ which included 34 pSS patients and 136 normal controls, found that the rates of spontaneous miscarriage, preterm birth and caesarean section were higher in pSS pregnancies. Additionally, the average birth weight and birth weight percentile of newborns were significantly lower in pSS pregnancies. The exploration of the impact of pSS diagnosis on pregnancy outcomes, whether before or during pregnancy, did not yield significant differences. In another study examining the impact of assisted reproductive technology (ART) on pregnancy outcomes in pSS patients, researchers found that preterm birth was the primary maternal APO, with no significant increase in fetal APO.^11^ Recent research on APOs in both SLE and pSS suggests that the most common APO in pSS pregnancies is miscarriage, and there is an association between prepregnancy complement levels and the development of APO.^12^

However, a recent prospective study in France showed different results, indicating no increased risk of APOs in women with pSS compared with the general population, even in cases of active pSS. Nonetheless, close monitoring is recommended for women with anti-phospholipid antibodies or anti-RNP antibodies.^13^ Thus, understanding the association between pSS and pregnancy is crucial.

While previous studies have investigated pSS pregnancies from various perspectives, the number of included pSS patients in these cohorts is generally limited, and the correlation between the disease status of pSS patients and different fetal outcomes has not been fully explored. Therefore, this multicentre retrospective study aims to understand differences in pregnancy and fetal outcomes between pSS patients and the general female population and to identify the risk factors leading to different pregnancy outcomes.

## Methods

### Study design and participants

#### Initial case screening

Preliminary screening was conducted on outpatient and inpatient cases of pregnant women with pSS who sought care in the Rheumatology and Obstetrics departments at the First Affiliated Hospital of the University of Science and Technology of China, Huangshan People’s Hospital, Huainan First People’s Hospital, Ma’anshan People’s Hospital and Chizhou People’s Hospital. The study cases were initially selected based on diagnostic keywords: “primary Sjögren’s syndrome,” “pregnancy” or “miscarriage” in the electronic medical record system from January 2015 to December 2022. The diagnosis of pSS was reviewed based on the 2012 ACR classification criteria or the 2016 American College of Rheumatology/European Alliance of Associations for Rheumatology classification criteria for Sjögren’s syndrome.^14 15^

#### Inclusion criteria

The following criteria were applied to the initially screened cases: (1) Inclusion of all cases with pSS and concurrent pregnancy. (2) Matching singleton pregnant women without any autoimmune diseases, who were treated during the same period, based on hospitalisation duration, as the control group. Each pSS case was matched with two singleton pregnant women without any autoimmune diseases. Each pregnancy was treated as an individual case, and patients could have multiple pregnancies included in the analysis.

#### Exclusion criteria

The exclusion criteria were as follows: (1) Cases with incomplete information. (2) Pregnancies in which the woman and fetus remained stable but were terminated due to the pregnant woman’s autonomy or self-determination. (3) Patients diagnosed with SLE, rheumatoid arthritis, spondyloarthritis, systemic sclerosis, inflammatory myopathy, antiphospholipid (APL) syndrome and mixed connective tissue diseases. (4) Pregnancies involving twins.

#### Population grouping based on pregnancy outcomes

Based on the fetal outcome, pSS patients were categorised into two main groups: a ‘normal pregnancy group’ and an ‘adverse pregnancy outcome group’.

The ‘normal pregnancy group’ in pSS was defined as pSS women who gave birth to healthy fetuses meeting the following criteria: gestational age between ≥37 and <42 weeks, birth weight between ≥2500 g and ≤4000 g, live birth of infants without malformations or diseases. The ‘adverse pregnancy outcome group’ in pSS included a variety of pregnancies that did not result in the birth of a healthy fetus, encompassing miscarriage (termination of pregnancies with a gestational age of less than 28 weeks), stillbirth (natural fetal demise in utero after 28 weeks of gestation or during delivery), preterm birth (delivery between 28 to less than 37 weeks of gestation), as well as low birth weight (birth weight less than 2.5 kg), morphological abnormalities, cardiac abnormalities, infections, metabolic abnormalities in newborns.

Further categorisation was based on the status of fetal live birth, leading to the division of pSS patients into two subgroups: a ‘fetal survival group’ and a ‘fetal loss group’. The ‘fetal survival group’ in pSS comprised pregnant women with pSS who delivered live-born neonates, although preterm birth, low birth weight and malformations might occur. The ‘fetal loss group’ consisted of pSS women who had experienced miscarriages and stillbirth. Ectopic pregnancies and molar pregnancies were excluded due to different underlying causes.

### Data collection

Data were reviewed through the electronic medical record system, and some missing data were obtained by contacting patients by telephone. The collected data included:

1. General Information: This encompassed disease duration, age, prepregnancy body mass index (BMI) (calculated as weight in kilograms divided by height in metres squared), mode of conception, gravidity, number of children, disease activity, comorbidities, serology, autoantibody spectrum and more. Prepregnancy indicators were derived from examinations and evaluations conducted during the last visit within 6 months before pregnancy. Late pregnancy indicators were based on prenatal examinations and evaluations after 28 weeks of pregnancy.
2. Pregnancy outcomes: This category covered various aspects, including the status of amniotic fluid, the presence or absence of threatened preterm labour (defined as regular contractions between 28 and less than 37 weeks of gestation without cervical dilatation), the type of fetal loss, the presence or absence of premature rupture of membranes, abnormal placenta (including placenta previa, placenta accreta, placenta adhesion, placental abruption, placental thrombosis, placental fibrin deposition, placental infarction, placental haematoma, placental haemorrhage, hydatidiform mole and chorioamnionitis), postpartum haemorrhage, postpartum intrauterine infection (defined as infection occurring in the uterine cavity, with main symptoms including fever, abdominal pain, vaginal purulent discharge, etc), and pregnancy complications (including gestational diabetes mellitus, pregnancy-induced hypertension, eclampsia/pre-eclampsia). Fetal loss included spontaneous miscarriage, therapeutic miscarriage and other outcomes (stillbirth, ectopic pregnancy, hydatidiform mole). Spontaneous miscarriage referred to the natural loss of a fetus while therapeutic miscarriage involved pregnancy termination due to disease activity or fetal abnormalities, including induced miscarriage and induced labour.
3. Fetal outcomes: These covered preterm birth, low birth weight (defined as birth weight less than 2.5 kg), fetal growth restriction (defined as deviation from expected fetal growth patterns due to various adverse factors, causing slower-than-normal growth), being small for gestational age (defined as birth weight below the 10th percentile or 2 SDs below the normal birth weight in the absence of placental insufficiency), neonatal brain injury (resulting from hypoxic-ischaemic brain damage during the perinatal period), neonatal asphyxia (failure to establish normal spontaneous breathing after birth leading to low oxygen levels), neonatal morphological malformations (morphological defects in certain body parts due to internal abnormal development), cardiac abnormalities (including elevated cardiac enzymes, atrial/ventricular septal defects, pulmonary hypertension and congenital heart conduction block), intrauterine distress (referring to acute or chronic hypoxia in utero endangering the health and life of the fetus), neonatal infection (comprising neonatal sepsis, neonatal infectious pneumonia, neonatal necrotising enterocolitis and neonatal omphalitis), electrolyte disturbances, acidosis and jaundice.
4. Medication use: This category documented the use of medications such as glucocorticoids, anticoagulants (including aspirin and/or low-molecular-weight heparin (LMWH)), and other immuno-anti-inflammatory therapies (eg, hydroxychloroquine (HCQ), cyclosporine, etc) before and during pregnancy.

### Statistical analysis

Continuous data results were presented as mean±SD, and categorical data were expressed as absolute values and percentages. Parameters following a normal distribution were analysed using t-tests while non-normally distributed parameters were assessed using the Mann-Whitney U test. χ^2^ tests were used to compare count-based parameters, and Fisher’s exact test was employed for frequencies below 1. Generalised linear regression was used to adjust for confounding factors in the analysis of pregnancy outcomes and live birth fetal outcomes, taking into account the presence of the same patient in the cohort with more than one pregnancy. For the prognostic factors analysis of APOs and fetal loss, univariate analysis was first employed to identify risk factors with statistically significant differences. Subsequently, these risk factors were included in binary logistic analysis to further screen for prognostic factors. Statistical significance was defined as a p value less than 0.05. IBM SPSS V.25.0 statistical software was employed for all analyses.

## Results

### Demographic data

**Figure 1:**
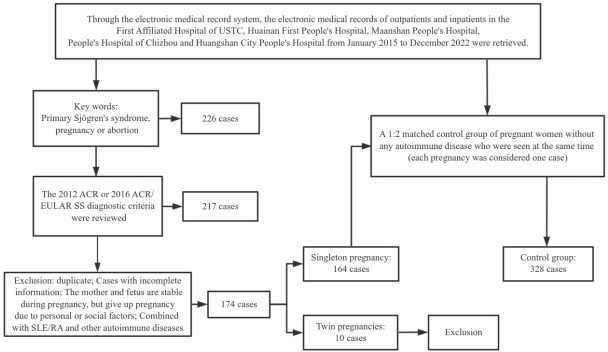
Study profile. ACR, American College of Rheumatology; EULAR, European Alliance of Associations for Rheumatology; SS, Sjögren’s syndrome.

This study initially retrieved 226 cases from the outpatient and inpatient medical record systems of several hospitals in the province. After further exclusions based on non-compliance with Sjögren’s syndrome classification criteria, duplicate cases, incomplete data, twin pregnancies and cases where pregnancy was terminated due to subjective factors (personal or family wishes), a total of 164 single pregnancies from 145 pSS patients were included. The control group consisted of 328 cases, all of whom had singleton pregnancies (figure 1).

The average age of the two groups was 31.98±4.55 and 31.83±4.52, with prepregnancy BMI of 21.14±2.04 and 21.21±1.7, respectively, and there were no statistically significant differences. In the pSS group, 37 cases (22.56%) used ART for conception while in the control group, only 3 cases (0.91%) did so, and this difference was statistically significant. In assisted reproduction, in vitro fertilisation and embryo transfer were the primary methods, with only one woman in the pSS group conceiving through intrauterine insemination. Furthermore, compared with the control group, there were no significant differences in the number of pregnancies in both groups, but fewer women in the pSS group had a history of childbirth (36.59% vs 54.88%, p<0.001). Regarding underlying diseases, including hypertension, diabetes, hepatitis B and others, there were no statistically significant differences between the two groups. However, more women in the pSS group had thyroid diseases (23.17% vs 1.22%, p<0.001), particularly hypothyroidism (19.51% vs 1.22%, p<0.001).

In terms of systemic involvement, 4.27% of the pSS group had renal tubular acidosis and 1.22% had interstitial pneumonia. In terms of serology, some of our data has been lost. Based on the existing data, 38.61% (39/101) had hypergammaglobulinaemia, 18.18% (12/66) had decreased complement 3 levels and there were no cases of decreased complement 4 levels. In the autoantibody spectrum, the proportions of positive anti-SSA52, anti-SSA60, anti-SSB, anti-RNP and anti-phospholipid antibodies were 86.08%, 85.44%, 42.41%, 6.96% and 13.25%, respectively. Regarding prepregnancy medication, 62.20% of pSS patients used glucocorticoid therapy, 60.98% used HCQ and 31.10% used aspirin. Additionally, 1.22% of patients received cyclosporine treatment due to low platelet counts (table 1).

**Table 1:** Preconception demographic data for pSS and control individuals

	pSS (n=164)	Controls (n=328)	t/χ^2^	P value
Age (years)*	31.98±4.55	31.83±4.52	0.34	0.736
Course of disease (months)*†	41.49±38.61	–		
BMI*‡	21.14±2.04 (145/164)	21.21±1.77	−0.43	0.670
Assisted reproductive conception§	37 (22.56%)	3 (0.91%)	68.589	<0.001
IVF-ET	36 (21.95%)	3 (0.91%)		
IUI	1 (0.61%)	0 (0%)		
Gravidity§				
1	51 (31.10%)	107 (32.62%)	0.117	0.733
2	43 (26.22%)	90 (27.44%)	0.082	0.774
3	37 (22.56%)	73 (22.26%)	0.006	0.939
>3	33 (20.12%)	58 (17.68%)	0.431	0.511
Parity§				
None	104 (63.41%)	148 (45.12%)	14.643	<0.001
Once	60 (36.59%)	180 (54.88%)	14.643	<0.001
Hypertension§	3 (1.83%)	0 (0%)		
Diabetes§	2 (1.22%)	0 (0%)		
Thyroid diseases§	38 (23.17%)	4 (1.22%)	67.474	<0.001
Hyperthyroidism	2 (1.22%)	0 (0%)		
Hypothyroidism	32 (19.51%)	4 (1.22%)	53.947	<0.001
Thyroiditis	4 (2.44%)	0 (0%)		
Kidney disease§	7 (4.27%)	0 (0%)		
Viral hepatitis B§	1 (0.61%)	3 (0.91%)	0.126	1.000
ILD§	2 (1.22%)	0 (0%)		
ESSDAI†‡§				
0–4	98/99 (98.99%)			
>4	1/99 (1.01%)			
Lymphocytopenia‡ (<1000/mm^3^)	10/116 (8.62%)			
Hypergammaglobulinaemia‡ (>16 g/L)	39/101 (38.61%)			
Low C3 level (<0.9 g/L)§‡	12/66 (18.18%)			
Low C4 level (<0.1 g/L)§‡	0/66 (0%)			
Autoantibody§‡				
Anti-Ro52	136/158 (86.08%)			
Anti-Ro60	135/158 (85.44%)			
Anti-La	67/158 (42.41%)			
Anti-DNA	0/158 (0%)			
Anti-RNP	11/158 (6.96%)			
APL	11/83 (13.25%)			
Prenatal medication§				
Glucocorticoid	102 (62.20%)			
Hydroxychloroquine	100 (60.98%)			
Ciclosporin	2 (1.22%)			
Aspirin	51 (31.10%)			

Data are mean±SD, n (%) or n/N (%). For variables with missing data, denominators are reported.

*Mean±SD.

†Patients diagnosed during pregnancy were not included.

‡There was data loss.

§Number with the percentages between brackets.

APL, antiphospholipid; BMI, body mass index; ESSDAI, EULAR Sjögren's Syndrome Disease Activity Index; ILD, Interstitial lung disease; IUI, intrauterine insemination; IVF-ET, in vitro fertilisation and embryo transfer techniques; n, number of deliveries; pSS, primary Sjögren’s syndrome.

### Pregnancy outcome

In the analysis of pregnancy outcomes, we employed logistic regression analysis to adjust for confounding factors. When comparing fetal outcomes, we observed that, in comparison to the control group, pregnant women in the pSS group were more likely to experience miscarriage, whether it was natural miscarriage (12.80% vs 1.52%, adjusted OR 11.335, p<0.001) or therapeutic miscarriage (6.10% vs 0.91%, adjusted OR 10.268, p<0.05). Natural miscarriages in the pSS group tended to occur earlier in pregnancy (gestational days at the time of miscarriage: 69.50±42.33 vs 155.43±30.48). Additionally, in the pSS group, a higher proportion of women had placental abnormalities detected during prenatal ultrasound examinations (14.63% vs 6.40%, adjusted OR 2.295, p<0.05). Other APOs, such as oligohydramnios, threatened preterm labour, premature rupture of membranes (preterm or full term), pre-eclampsia/eclampsia, gestational hypertension and gestational diabetes, showed no statistically significant differences between the two groups (table 2).

**Table 2:** Pregnancy outcome for pSS cases and control individuals

	pSS (n=164)	Controls (n=328)	Adjusted OR	95% CI	P value
Fetal loss*	36 (21.95%)	12 (3.66%)			
Spontaneous miscarriage	21 (12.80%)	5 (1.52%)	11.335	(4.128 to 31.123)	<0.001
Duration of spontaneous miscarriage (day)†	69.50±42.33	155.43±30.48			
Therapeutic miscarriage	10 (6.10%)	3 (0.91%)	10.268	(2.646 to 39.846)	0.001
Other‡	5 (3.05%)	4 (1.22%)	2.319	(0.572 to 9.412)	0.239
Oligohydramnios*	26 (15.85%)	35 (10.67%)	1.672	(0.936 to 2.989)	0.083
Eclampsia/pre-eclampsia*	2 (1.22%)	5 (1.52%)	0.319	(0.042 to 2.430)	0.270
Threatened preterm birth*	7 (4.27%)	3 (0.91%)	3.896	(0.925 to 16.412)	0.064
Premature rupture of membranes*	23 (14.02%)	56 (17.07%)			
Premature rupture of membranes in preterm	16 (9.76%)	50 (15.24%)	0.728	(0.391 to 1.356)	0.317
Premature rupture of membranes in full term	7 (4.27%)	6 (1.83%)	1.671	(0.502 to 5.564)	0.403
Placental abnormalities on ultrasound*	24 (14.63%)	21 (6.40%)	2.295	(1.182 to 4.457)	0.014
Placenta previa	6 (3.66%)	6 (1.83%)			
Placental abruption	1 (0.61%)	3 (0.91%)			
Hydatid mole	1 (0.61%)	0 (0%)			
Placenta implantation	3 (1.83%)	1 (0.30%)			
Placental adherence	9 (5.49%)	4 (1.22%)			
Placental thrombosis	1 (0.61%)	0 (0%)			
placental fibrin deposition	6 (3.66%)	0 (0%)			
Placental infarction	1 (0.61%)	0 (0%)			
Placental haematoma or bleeding	5 (3.05%)	5 (1.52%)			
Chorioamnionitis	4 (2.44%)	1 (0.30%)			
Veil-shaped placenta	2 (1.22%)	3 (0.91%)			
Racquet-shaped placenta	2 (1.22%)	1 (0.30%)			
Pregnancy-induced hypertension*	6 (3.66%)	6 (1.83%)	2.367	(0.624 to 8.978)	0.205
Gestational diabetes mellitus*	18 (10.98%)	27 (8.23%)	1.339	(0.673 to 2.666)	0.406

Data were analysed by generalised linear regression method logistic.

*Number with the percentages between brackets.

†Mean±SD.

‡Including stillbirth, ectopic pregnancy, hydatidiform mole.

n, number of deliveries; pSS, primary Sjögren’s syndrome.

Furthermore, we conducted a detailed review of the reasons for therapeutic miscarriages in the 10 cases from the pSS group. Due to the high risk of fetal cardiac abnormalities in pSS, routine fetal cardiac ultrasound examinations and regular fetal heart monitoring were performed around the 26th week of pregnancy for patients with anti-SSA antibodies. One patient with pSS underwent artificial miscarriage due to the discovery of complete heart block (CHB) in a prenatal echocardiogram, which resulted in intrauterine fetal death after ineffective treatment. Other reasons for therapeutic miscarriages included severe fetal malformation in one case, fetal distress due to umbilical blood flow loss in one case and six cases where miscarriage was necessary due to maternal disease activity (mainly manifested as refractory severe thrombocytopaenia or widespread purpura) requiring potentially teratogenic medications.

In this study, the number of stillbirth cases in the pSS group was minimal, with only one case undergoing therapeutic induction due to intrauterine fetal demise occurring after 37 weeks of gestation. Meanwhile, in the HC group, there were two cases of fetal demise, occurring at 29 and 34 weeks of gestation, respectively. There was no statistically significant difference in the occurrence of stillbirth between the two groups.

### Live birth fetal outcomes

To clarify the differences in live births between the pSS and control groups, we compared data from 128 pregnancies in the pSS group and 316 pregnancies in the control group. We used logistic regression analysis to control for confounding factors. We found that, compared with the control group, caesarean section was the predominant mode of delivery for newborns in the pSS group (78.44% vs 42.41%, adjusted OR 3.627, p<0.001). Newborns with suspected cardiac abnormalities, especially those with symptoms like cyanosis, persistent restlessness, the presence of heart murmurs or those requiring admission to the paediatric department, underwent serological and cardiac ultrasound examinations. Newborns without indications for further cardiac ultrasound examination were considered to have normal hearts. The results showed a higher incidence of cardiac abnormalities in the pSS group (27.34% vs 12.03%, adjusted OR 3.121, p<0.05), particularly elevated cardiac enzymes (19.53% vs 10.76%) and atrioventricular septal defects (13.28% vs 1.58%). No newborns in either group clinically manifested CHB.

Regarding preterm birth, low birth weight, neonatal asphyxia, fetal growth restriction/small for gestational age, intrauterine distress, neonatal brain injury, external morphological abnormalities, neonatal infections, acidosis, electrolyte disturbances and hypoglycaemia, there were no statistically significant differences between the two groups. Furthermore, some adverse neonatal findings were observed during haematological testing after admission to the neonatal department. Five cases had anti-SSA60/SSA52 antibodies, and 26 newborns had normal electrocardiograms without any significant abnormalities detected. On the maternal side, pSS women were more prone to postpartum uterine cavity infections (2.34% vs 0.63%, adjusted OR 11.187, p<0.05), but there were no significant statistical differences between the two groups in terms of postpartum haemorrhage. Additionally, the proportion of hospitalisation for newborns in the pSS group showed a relative increase, although there was no statistically significant difference. The reasons for hospitalisation were often associated with preterm birth, intrauterine distress, neonatal infections, amniotic fluid contamination and other factors (table 3).

**Table 3:** Adverse outcomes of live birth fetuses

	pSS (n=128)	Controls (n=316)	Adjusted OR	95% CI	P value
Caesarean section*	94 (78.44%)	134 (42.41%)	3.627	(2.172 to 6.059)	<0.001
Premature birth*	17 (13.28%)	16 (5.06%)	2.227	(0.623 to 7.956)	0.218
Gestational age (day)†	240.07±17.80	240.31±17.23			
Induced premature births	14 (10.94%)	11 (3.48%)			
Underweight*	14 (10.94%)	13 (4.11%)	0.767	(0.160 to 3.676)	0.740
IUGR/SGA*	3 (2.34%)	4 (1.27%)	1.561	(0.152 to 16.031)	0.708
Postpartum intrauterine infection*	3 (2.34%)	2 (0.63%)	11.187	(1.025 to 122.123)	0.048
Postpartum haemorrhage*	5 (3.91%)	8 (2.53%)	0.780	(0.185 to 3.286)	0.735
Neonatal asphyxia*	3 (2.34%)	10 (3.16%)	0.243	(0.041 to 1.450)	0.121
Intrauterine distress*	16 (12.50%)	23 (7.28%)	1.225	(0.515 to 2.910)	0.646
Neonatal brain injury*	11 (8.59%)	19 (6.01%)	1.009	(0.327 to 3.114)	0.988
Superficial morphological malformation*	1 (0.79%)	2 (0.63%)	0.684	(0.048 to 9.746)	0.779
Cardiac abnormalities*	35 (27.34%)	38 (12.03%)	3.121	(1.333 to 7.307)	0.009
Elevated cardiac enzymes	25 (19.53%)	34 (10.76%)			
Atrial/ ventricular septal defects	17 (13.28%)	5 (1.58%)			
CHB	0 (0%)	0 (0%)			
Pulmonary hypertension	1 (0.79%)	1 (0.32%)			
Bovine aortic arch	1 (0.79%)	0 (0%)			
Neonatal infection*	23 (17.97%)	31 (9.81%)	1.030	(0.420 to 2.527)	0.949
Neonatal acidosis*	2 (1.56%)	5 (1.58%)	0.535	(0.075 to 3.824)	0.533
Electrolyte disorder of newborn*	1 (0.79%)	2 (0.63%)	2.193	(0.103 to 46.684)	0.615
Neonatal jaundice*	14 (10.94%)	21 (6.65%)	1.330	(0.555 to 3.185)	0.522
Neonatal hospital admission*	45 (35.16%)	66 (20.89%)	1.132	(0.489 to 2.620)	0.772

Data were analysed by generalised linear regression method logistic.

*Number with the percentages between brackets.

†Mean±SD.

CHB, complete heart block; IUGR/SGA, intrauterine growth restriction/small for gestational age; n, number of deliveries.

### Analysis of risk factors for APOs

In the analysis of risk factors for APOs, we compared APOs in pSS patients with normal pregnancies from multiple perspectives. There were no statistically significant differences between the two groups in terms of patient age, disease duration, time of pSS diagnosis (prepregnancy or during pregnancy), history of miscarriages, method of conception (natural or ART), pregnancy complications, medications during pregnancy and autoantibodies (all p>0.05). However, when comparing complement levels, we found that the C4 levels in the late pregnancy period were significantly lower in the group with APOs compared with the normal pregnancy group (0.21±0.06 vs 0.27±0.08, p<0.05), but there were no significant differences in complement levels at other time points (table 4).

**Table 4:** Comparison of maternal pSS with different fetal outcomes

	Normal pregnancy (n=68)	Adverse pregnancy outcomes (n=96)	t/χ^2^	P value	Fetal survival (n=128)	Fetal loss* (n=32)	t/χ^2^	P value
Age (years)†	31.47±4.06	32.33±4.86	−1.235	0.219	32.02±4.23	31.75±5.73	0.303	0.762
Course of disease (months)†‡	40.83±33.70	41.97±42.04	−0.863	0.179	42.96±38.96	30.24±29.06	1.591	0.114
pSS diagnosed before pregnancy§	57 (83.82%)	82 (85.42%)	0.078	0.780	108 (84.38%)	27 (84.38%)	0.000	1.000
History of miscarriage§	45 (66.18%)	55 (57.29%)	1.321	0.251	77 (60.16%)	17 (53.13%)	0.522	0.470
ART conception§	14 (20.59%)	23 (23.96%)	0.259	0.611	30 (23.43%)	7 (21.88%)	0.035	0.851
Accompany diseases during pregnancy§								
Hypertension	1 (1.47%)	5 (5.21%)	0.695	0.404	6 (4.69%)	0 (0%)	0.530	0.466
Diabetes	7 (10.29%)	11 (11.46%)	0.055	0.814	15 (11.72%)	2 (6.25%)	0.333	0.564
Thyroid disease	17 (25.00%)	21 (21.88%)	0.218	0.640	33 (25.78%)	4 (12.50%)	2.540	0.111
Viral hepatitis B	1 (1.47%)	0 (0%)	/	0.415	1 (0.78%)	0 (3.13%)	/	1.000
Pregnancy medicine§								
GC	47 (69.12%)	76 (79.17%)	2.144	0.143	99 (77.34%)	22 (68.75%)	1.026	0.311
HCQ	48 (70.59%)	69 (71.88%)	0.015	0.902	96 (75.00%)	19 (59.38%)	3.092	0.079
CyA	1 (1.47%)	8 (8.33%)	2.412	0.120	6 (4.69%)	3 (9.38%)	0.361	0.548
LMWH	26 (38.24%)	31 (32.29%)	0.620	0.431	51 (39.84%)	6 (18.75%)	4.967	0.026
ASA	36 (52.94%)	42 (43.75%)	1.348	0.246	68 (53.13%)	10 (31.25%)	4.903	0.027
Serological indicators† ¶								
C3 (before pregnancy)	1.11±0.20 (23/68)	1.02±0.16 (43/96)	1.877	0.065	1.06±0.17 (47/128)	1.01±0.18 (15/32)	0.919	0.362
Low C3 level (before pregnancy) (<0.9 g/L)	4/23 (17.40%)	8/43 (18.60%)	0.000	1.000	8/47 (17.02%)	3/15 (9.38%)	0.000	1.000
C4 (before pregnancy)	0.25±0.08 (23/68)	0.22±0.06 (43/96)	1.235	0.221	0.23±0.08 (47/128)	0.24±0.05 (15/32)	−0.364	0.717
Low C4 level (<0.1 g/L)	0/23 (0%)	0/43 (0%)			0/47 (0%)	0/15 (0%)		
C3 (late pregnancy**)	1.29±0.23 (27/68)	1.21±0.21 (36/96)	1.458	0.150				
Low C3 level (late pregnancy**) (<0.9 g/L)	4/27 (14.81%)	1/36 (2.78%)	1.634	0.201				
C4 (late pregnancy**)	0.27±0.08 (27/68)	0.21±0.06 (36/96)	3.003	0.004				
Low C4 level (<0.1 g/L)	0/27 (0%)	1/36 (2.7%)	/	1.000				
Autoantibody§								
Anti-Ro52	57/64 (89.06%)	79/94 (84.4%)	0.801	0.371	105/122 (86.07%)	27/32 (84.38%)	0.000	1.000
Anti-Ro60	56/64 (87.50%)	78/94 (82.98%)	0.604	0.437	105/122 (86.07%)	25/32 (78.13%)	0.686	0.407
Anti-LA	30/64 (46.88%)	37/94 (39.36%)	0.880	0.348	52/122 (42.62%)	12/32 (37.50%)	0.274	0.601
APL	1/30 (3.33%)	10/53 (18.87%)	2.783	0.095	9/60 (15.00%)	2/21 (9.52%)	0.000	1.000

Data are mean±SD, n (%) or n/N (%). For variables with missing data, denominators are reported.

*Adjusted data after excluding ectopic pregnancies and hydatidiform moles.

†Mean±SD.

‡Patients diagnosed during pregnancy were not included.

§Number with the percentages between brackets.

¶There was data loss.

**After 28 weeks of pregnancy.

APL, antiphospholipid antibody; ART, assisted reproductive technology; ASA, aspirin; CyA, ciclosporin A; GC, glucocorticoid; HCQ, hydroxychloroquine; LMWH, low-molecular-weight heparin; n, number of deliveries; pSS, primary Sjögren’s syndrome.

In the analysis of risk factors for fetal loss, we analysed and compared the same factors as mentioned above. We found that, compared with the fetal loss group, the fetal survival group had a higher use of (39.84% vs 18.75%, p<0.05) and aspirin (53.13% vs 31.25%, p<0.05) for anticoagulation therapy. On the other hand, the fetal loss group had a higher rate of interstitial pneumonia (6.25% vs 0%, p<0.05). Further binary logistic regression analysis of these different factors and possible related factors (p values <0.1) revealed that the use of LMWH was an independent protective factor for fetal survival (table 5).

**Table 5:** Multivariate logistic regression analysis of fetal survival and fetal loss groups

	Univariate			Multivariate*		
	OR	95% CI	P value	OR	95% CI	P value
HCQ	0.615	(0.368 to 1.030)	0.079	0.683**†**	/	/
LMWH	0.348	(0.134 to 0.906)	0.026	0.362	(0.139 to 0.945)	0.038
ASA	0.401	(0.176 to 0.914)	0.027	0.694**†**	/	/

*Due to the limited number of independent variables, binary logistic regression with forward: LR method was employed for analysis.

†Binary logistic regression with enter method was employed for analysis.

ASA, aspirin; HCQ, hydroxychloroquine; LMWH, low-molecular-weight heparin.

## Discussion

Previous studie have suggested that autoimmune diseases do not detrimentally affect fertility.^16^ In contrast, our investigation reveals that 22.56% of women with pSS necessitate assisted reproductive techniques for conception, a markedly higher proportion compared with the control group. This implies that natural conception might pose greater challenges for women afflicted with pSS. Furthermore, there was no significant difference in the number of pregnancies between the two groups, yet women with pSS had fewer live births, indicating a higher rate of miscarriages among this population and highlighting the elevated risk associated with pregnancy in pSS patients. Regarding prepregnancy comorbidities, our observations showed a higher prevalence of thyroid diseases, primarily hypothyroidism, in the pSS group, which could be attributed to the systemic involvement of pSS itself. As for disease activity, we only included women diagnosed with pSS prior to pregnancy, and 98.99% of these patients had stable disease status, a result of close monitoring during the prepregnancy period.

In our analysis of pregnancy outcomes, we noted a higher incidence of placental abnormalities detected during prenatal ultrasound examinations among pregnant women diagnosed with pSS. Nevertheless, no statistically significant disparities were observed in placental-related conditions, including preterm labour and pregnancy-induced hypertension, between the pSS group and the control cohort. Regarding the occurrence of pre-eclampsia or gestational hypertension, women with pSS exhibited similar rates compared with their healthy counterparts. Furthermore, our investigation revealed a heightened prevalence of miscarriages in the pSS cohort relative to the control group. This finding aligns with the observations made by Ballester *et al*, who documented an elevated rate of spontaneous miscarriages in pSS patients in their study involving 59 pregnancies.^17^ Within our dataset, both spontaneous and therapeutic miscarriages were more prevalent in the pSS group. Additionally, our data indicated an earlier onset of spontaneous miscarriage in individuals with pSS. This phenomenon may be attributed to heightened vigilance from both healthcare providers and patients regarding pregnancies in individuals with pSS, leading to more prompt detection of early miscarriages.

When comparing the outcomes of live births, both the pSS group and the control group did not exhibit significant differences in terms of preterm births. This is consistent with the recent findings by Fierro *et al*.^12^ However, Chan *et al*, in their investigation of pregnant women in Taiwan, found that pregnant women with pSS had a significantly increased risk of pulmonary oedema and shock, along with a higher prevalence of low birth weight (<2500 g), small for gestational age infants and fetal distress.^18^ In our study, while the risk of postpartum uterine cavity infection was higher in pSS pregnant women, there were no significant differences in low birth weight, small for gestational age or fetal distress compared with healthy women. In terms of medications related to pSS, we observed a high frequency of steroid and HCQ use both before and during pregnancy. The utilisation of glucocorticoids may be associated with the patient’s previous disease activity, but with long-term treatment, the disease tends to stabilise and glucocorticoids are still employed as maintenance drugs. In this study, the increased incidence of postpartum intrauterine infection in the pSS group may be linked to the lowered immunity resulting from glucocorticoid application. However, it is crucial to note that the total number of intrauterine infection cases was relatively low (five cases in total, three in the pSS group and two in the control group), and the possibility of bias cannot be completely ruled out.

Historically, scholars have posited that pSS exerts its primary impact on pregnancy through neonatal lupus, with congenital heart block being the most significant complication of neonatal lupus due to its permanence. Autoimmune CHB is an exceedingly rare condition, with an estimated incidence of 1 in 20 000 live births.^19^ In a comprehensive study by Brito-Zerón *et al*, they reported 44 cases of autoimmune CHB in 49 infants born to pSS mothers.^20^ Among five recurrent CHB cases, four had positive anti-RO and anti-LA antibodies. Of the 35 surviving infants with CHB (71%), 5 (14%) displayed characteristics of neonatal lupus. However, in our dataset, we only identified one case of congenital heart block in a pregnant woman during prenatal screening, and a therapeutic miscarriage was performed in this case. Previous literature has also reported cases of pSS combined with CHB that were unresponsive to treatment, with the most opting for induced abortion (85.7%, 12/14).^20^

In our research, the more common cardiac manifestations observed were elevated cardiac enzymes and atrial/ventricular septal defects. Elevated cardiac enzymes are likely attributed to the high sensitivity of cardiac enzymes and may be associated with factors such as fetal ischaemia, hypoxia or infection during intrauterine or delivery-related processes. While congenital atrial/ventricular septal defects are infrequently reported in autoimmune diseases, they are generally associated with chromosomal abnormalities in the general population.^21^ Furthermore, there is a higher risk of atrial/ventricular septal defects in cases where mothers have chronic conditions such as chronic hypertension, epilepsy/migraines or paroxysmal supraventricular tachycardia.^22^ The reasons for the increased incidence of atrial/ventricular septal defects in pSS remain unclear and may involve factors such as medication use or chronic viral infections, potentially contributing to intrauterine hypoxia.

We also observed a significantly higher rate of caesarean section in pregnant women with pSS. Although previous studies have reported similar findings, our study did not identify a higher proportion of older mothers or low birth weight infants in the pSS group.^23^ We speculate that the high caesarean section rate may be aimed at shortening the duration. However, the high rate of caesarean section still entails some unnecessary risks. Therefore, it is essential to exercise strict criteria when choosing the mode of delivery, particularly in determining the indications for caesarean section. Research has indicated that the use of HCQ during pregnancy significantly reduces the incidence of pre-eclampsia, early-onset pre-eclampsia and mid-to-late pregnancy fetal loss in individuals with autoimmune diseases.^24^ HCQ, currently recognised as a non-specific anti-inflammatory agent, functions by accumulating in the cell cytoplasm, thereby inhibiting autophagy and regulating intracellular homeostasis. It can also exert anti-inflammatory effects by inhibiting toll-like receptors, inflammatory factors, and complement responses.^25^ Additionally, HCQ’s antithrombotic properties have been observed in clinical studies, although the precise mechanisms remain unclear.^26^ Based on early and limited data, the 2020 ACR guidelines for Reproductive Health Management in Rheumatic and Musculoskeletal Diseases recommend the use of HCQ during pregnancy for all patients positive for anti-SSA/SSB antibodies.^27^ In this study, over half of the pregnant women were on HCQ treatment, yet we did not observe any preventive effect of HCQ on APOs or fetal loss. Furthermore, in this research, anticoagulant medications (aspirin and low-molecular-weight heparin) were widely employed in pSS pregnancies. While the specific indications for their use were not investigated, it is speculated that they may be related to factors such as positivity for APL antibodies, coagulation abnormalities, a history of recurrent ART implantation failures and previous APOs. In the analysis of fetal loss risk factors, we found that the use of aspirin and low-molecular-weight heparin was more common in the fetal survival group. Further multifactorial analysis revealed that the use of low-molecular-weight heparin is an independent protective factor for fetal survival. This may be due to the potential of low-molecular-weight heparin to improve hypercoagulable states, thereby ensuring adequate placental blood supply and creating a favourable environment for fetal survival while its direct impact on the fetus, which does not cross the placental barrier significantly, is minimal. Additionally, heparin can promote angiogenesis and support vascular crosstalk between trophoblasts and endothelial cells.^28^ In the presence of APL, LMWH can counteract inflammation in the trophoblast but induces an anti-angiogenic response.^29^ LMWH enhances the secretion of placental growth factors from endothelial cells.^30^ This may explain why LMWH treatment prevents early pregnancy loss. However, there is currently no conclusive evidence to recommend heparin for improving pregnancy outcomes in women. In a randomised trial, women with at least two prior pregnancy losses were randomly assigned to receive aspirin combined with LMWH, aspirin alone or placebo, with no significant difference in live birth rates observed among the three groups.^31^ Another meta-analysis also indicated that aspirin and/or LMWH did not increase the likelihood of live birth in patients with recurrent miscarriage.^32^ Therefore, reducing unnecessary use of LMWH is necessary, and preconception haemodynamic assessment may help identify high-risk women with placental diseases who require prophylactic anticoagulant therapy.

Recent studies on pregnant women with pSS have demonstrated a significant correlation between low C4 levels before pregnancy and APOs.^12^ However, in our study, we did not observe an association between prepregnancy complement levels and APO. Conversely, when comparing normal pregnancy with APOs in the context of pSS, we found that C4 levels in the late stages of normal pregnancies were significantly higher than those in the APO group, despite both groups having generally normal C4 levels. Therefore, we do not consider it to be a clinically relevant variable.

## Conclusion

In summary, compared with regular pregnancies, women with pSS encounter increased difficulty conceiving, heightened risks of miscarriage, a greater likelihood of fetal abnormalities (especially cardiac abnormalities), and a higher incidence of postpartum complications. Regular monitoring of the disease, screening for fetal anomalies and comprehensive health surveillance throughout the entire pregnancy in pSS patients are imperative. A multidisciplinary approach involving rheumatology, obstetrics and paediatrics will likely become the standard for managing pSS pregnancies. In our study, we found that LMWH anticoagulant therapy is an independent protective factor for fetal survival. However, we did not identify independent risk factors for APOs, which may be attributed to our sample size. Further research involving a larger, multicentre, prospective study is warranted to better elucidate the impact of pSS on pregnancy outcomes.

## Data Availability

Data are available on reasonable request.
